# Metabolic innovations towards the human lineage

**DOI:** 10.1186/1471-2148-8-247

**Published:** 2008-09-09

**Authors:** Shiri Freilich, Leon Goldovsky, Christos A Ouzounis, Janet M Thornton

**Affiliations:** 1 The European Bioinformatics Institute, EMBL Cambridge Outstation, Wellcome Trust Genome Campus, Cambridge CB10 1SD, UK; 2 School of Computer Science, Tel Aviv University, Tel Aviv 69978, Israel; 3 School of Medicine, Tel Aviv University, Tel Aviv 69978, Israel; 4 Computational Genomics Unit, Institute of Agrobiotechnology, Center for Research and Technology Hellas, GR-57001 Thessalonica, Greece; 5 Centre for Bioinformatics, School of Physical Sciences and Engineering, King's College London, Strand, London WC2R 2LS, UK

## Abstract

**Background:**

We describe a function-driven approach to the analysis of metabolism which takes into account the phylogenetic origin of biochemical reactions to reveal subtle lineage-specific metabolic innovations, undetectable by more traditional methods based on sequence comparison. The origins of reactions and thus entire pathways are inferred using a simple taxonomic classification scheme that describes the evolutionary course of events towards the lineage of interest. We investigate the evolutionary history of the human metabolic network extracted from a metabolic database, construct a network of interconnected pathways and classify this network according to the taxonomic categories representing eukaryotes, metazoa and vertebrates.

**Results:**

It is demonstrated that lineage-specific innovations correspond to reactions and pathways associated with key phenotypic changes during evolution, such as the emergence of cellular organelles in eukaryotes, cell adhesion cascades in metazoa and the biosynthesis of complex cell-specific biomolecules in vertebrates.

**Conclusion:**

This phylogenetic view of metabolic networks puts gene innovations within an evolutionary context, demonstrating how the emergence of a phenotype in a lineage provides a platform for the development of specialized traits.

## Background

Comparative genomics studies provide a global view of the innovations in the gene repertoire within a lineage, and they are most useful in identifying novel gene families, or families that have been massively expanded. The comparative analysis of gene families and their lineage-specific expansions thus provide fundamental insights into the innovations of a gene repertoire for a taxonomic group under investigation. In many cases, the evolutionary expansion of a family, in terms of numbers of genes within a species, can be directly linked to a characteristic trait of the lineage. For example, genes that have been characterized as eukaryotic-specific are mostly involved in the organization of DNA within the nucleus, the compartmentalization of the cytoskeleton, membrane transport, the control of the cell-cycle, various regulatory processes, and RNA splicing [1]. Gene families that have massively expanded during the evolution of metazoa include many transcription factors (participating in spatial and developmental differentiation), signaling molecules (acting as mediators of inter-cellular communication), and adhesion molecules (integrating cell types into tissues) [2]. Many vertebrate-specific genes and gene families are involved in processes related to defense and immunity (e.g., the immunoglobulins), as well as the nervous system (e.g., olfactory receptors) [3].

A limitation of this sequence-driven approach is that subtle changes, which do not necessarily involve massive expansions, cannot, in many cases, be deciphered in such comparative studies. Nonetheless, this type of change might reflect an important adaptation of a species to its environment that cannot be readily detected by sequence information alone. Examples include the adaptation of the colobine monkey RNASE1B to the pH environment in its small intestine, probably as a response to its unique diet [4]. Another example is the origin of a prokaryotic NADP-dependent IDH (isocitrate dehydrogenase) from an ancestral NAD-dependent IDH, as a response to metabolic demands for growth on acetate [5]. These differences are virtually indistinguishable at the sequence level.

For enzymatic proteins, the well-studied relationship between sequence and function allows us to translate genomic information into relatively concise functional predictions [6-8]. Therefore, for this class of proteins, we can easily construct a high-resolution set of functional predictions for a variety of fully-sequenced species. The availability of several enzymatic-oriented annotation schemes permits a sensitive study, where the distribution of reactions (rather than sequences) in different lineages can be compared. Prominent enzymatic classification resources include schemes such as the Enzyme Commission (EC) scheme [9], which links sequence and enzymatic reaction, and metabolic databases [10], such as the KEGG database [11], which classify enzymatic reactions into metabolic pathways. As previously shown, the use of these databases can provide valuable insights into the relationship between functional innovations in the metabolic-reaction repertoire of a species and lineage-specific adaptation [12,13]. The classification of the homologous NAD-dependent IDH and the NADP-dependent IDH into distinct EC categories (EC 1.1.1.41 and 1.1.1.42, respectively) points to the potential of this approach for identifying lineage-specific functional innovations that did not necessarily involve massive gene expansion.

Here, we focus on the examination of lineage-specific innovations during the evolution of the human metabolic network. Primarily, we aim to gain a better understanding of the relationship between the evolution of the human metabolic set and the development of new, tissue-specific pathways. Several factors indicate that tissue differentiation is, at least in part, accompanied by tissue specialization of metabolism. First, most of the enzymatic genes have specialized expression patterns and they are not globally expressed across mammalian tissues [14]. Second, although many of the human enzymes are conserved between human and prokaryotes, there are still many human enzymes which are specific to eukaryotic, metazoan, and vertebrate species [15]. The variation of these enzymes during the evolution of the lineage leading to human has not been investigated in detail. We used the KEGG pathway database to describe the metabolic pathways in human, to characterize their evolutionary origin, and to study the structure of the network these pathways form. This function-based analysis performed here enables us to use genomic data in order to identify lineage-specific functional innovations, understand their contribution in the wider context of metabolic networks, and outline the gradual process of species evolution. The main novelty in this function-based analysis is the provision of a global view on the evolution of the mammalian metabolic-network.

## Methods

### Retrieving a set of the human metabolic reactions

The distribution of enzymatic reactions (EC numbers) in species was retrieved from the KEGG database [11,16] (Release 41.1). For each reaction, KEGG provides predictions for its occurrence in a collection of 540 species (including 422 bacterial species, 32 archaeal species, and 86 eukaryotic species). The species distribution of each reaction was processed by parsing the 'enzyme' file (downloaded from ftp://ftp.genome.jp/pub/kegg/ligand/). In total, KEGG provides gene assignment (in at least a single species) for 2,272 reactions, out of which 971 reactions are predicted to occur in human (4 digit EC numbers).

### Retrieving a set of the human metabolic pathways and linking them to form a network

The classification of reactions into pathways was retrieved from the KEGG database. KEGG describes diagrams of 142 metabolic pathways, where each pathway is composed of a collection of enzymatic reactions. The pathway-distribution of each reaction was processed by parsing the 'enzyme' file.

In total, the 971 human reactions are distributed among 117 pathways. The erroneous inclusion of the photosynthesis pathway in this set of 117 pathways indicates that the occurrence of a pathway in a species cannot be simply inferred from the presence of one reaction which participates in that pathway. In the photosynthesis pathway, some of the participating reactions, such as ATP synthase (EC 3.6.3.14), also participate in other pathways, such as the oxidative phosphorylation pathway. ATP synthase is a universal enzyme which is widespread among species and domains of life, but it takes part in different pathways across various lineages. This example points to two limiting factors, arising from the structure of the KEGG pathway diagrams, which make the inference of a species' pathway repertoire based on the species' reaction repertoire a highly non-trivial exercise. First, the reactions are not necessarily unique to a single pathway, and about half of the reactions occur in multiple pathways. Second, the pathways in KEGG are reference pathways – i.e., they are a composite collection of all relevant reactions found in all species [17]. Since the pathways in KEGG provide a collective, rather than a species-specific, view of a pathway and further, some reactions are shared between several pathways, the occurrence of a reaction in a species is not sufficient to infer the occurrence of a pathway in a species.

To address this problem and focus on those pathways which are pertinent to our analysis, we have considered only pathways where at least a single human reaction is either pathway-specific, or vertebrate-specific: the rationale being that each of those reactions is thus either biochemically specific or taxonomically relevant, respectively (or indeed, both). We have also excluded pathways where less than three participating reactions were found in the human species. This results in a set of 78 pathways detected in human to which 652 human enzymes are assigned.

The KEGG scheme provides information about the links between different pathway maps (e.g., the glycolysis pathway is linked with the pentose phosphate pathway). Pathway A and pathway B are linked if a product of a reaction from pathway A is the substrate of a successive reaction from pathway B. The information was retrieved from the .map files downloaded from ftp://ftp.genome.jp/pub/kegg/pathway/map. We used this information to construct the network representation of the metabolic pathways in human. Each pathway is a node in the network and is connected to other pathways according to the links in the KEGG diagram.

### Classification of human reactions and pathways into phylogenetic groups

All human reactions considered here were further classified into four groups, describing the putative lineage where those reactions have emerged. This classification was performed according to predictions in KEGG for the distribution of reactions in the collection of species (as described earlier in the section 'Retrieving a set of the human metabolic reactions'). Vertebrate-specific reactions are those human reactions which are predicted to be limited to vertebrates; metazoan-specific reactions are those human reactions which are predicted to occur only in metazoan species (including metazoan species other than vertebrates); eukaryotic-specific reactions are those human reactions which are predicted to be limited to eukaryotes (including eukaryotic species other than metazoa); universal reactions are human reactions which are widely distributed in prokaryotic species (found in more than 10 prokaryotes, either Bacteria or Archaea).

For each pathway, we have estimated its lineage of origin according to the phylogenetic age of its reaction-members, where pathways were assigned according to the classification of their most ancient unique reaction.

## Results

### The phylogenetic origin of metabolic reactions in human

The distribution of the human reactions from the 78 selected pathways into the phylogenetic groups is shown in Table 1. Reassuringly, the distribution pattern obtained from KEGG is similar to the pattern previously obtained using a different method based on in-house sequence comparisons [15]. Both classifications indicate that about half of the vertebrate reactions are predicted to have a pre-eukaryotic origin (i.e. universal reactions, according to our definition – see **Methods**), forming the previously reported extensive conserved core of metabolic enzymes common to archaea, bacteria and eukaryotes [15,18] (Table 1). The remaining reactions are innovations, specific to more recent lineages (Table 1). The large fraction of vertebrate-specific reactions, compared to metazoan-specific reactions, is in agreement with a recent study reporting the extensive gain of metabolic reaction, following vertebrata-invertebrate split [19]. Here, we have used the KEGG pathway scheme in order to obtain a better view of the functional contribution of the lineage-specific reactions.

**Table 1:** The distribution of human reactions from the 78 selected pathways within metabolic super-pathways.

KEGG super-pathway	Total number of reactions	Universal reactions	Eukaryotic-specific reactions	Metazoan-specific reactions	Vertebrate-specific reactions
All	652(301)	367(168)	128(67)	35(16)	122(50)

Carbohydrate Metabolism	160(61)	114(41)	25(9)	7(4)	14(7)
Energy Metabolism	30(9)	23(6)	3(2)	4(1)	4(1)
Lipid Metabolism	138(70)	54(19)	45(29)	3(2)	36(20)
Nucleotide metabolism	72(38)	61(33)	8(4)	0(0)	3(1)
Amino acid metabolism	168(61)	120(35)	20(9)	6(5)	22(12)
Metabolism of other amino acids	36(4)	30(3)	3(0)	1(1)	2(0)
Glycan biosynthesis & metabolism	87(7)	14(1)	20(4)	16(1)	37(1)
Metabolism of cofactors and Vitamins	80(48)	57(30)	8(8)	5(3)	10(7)
Biosynthesis of secondary metabolites	5(0)	2(0)	0(0)	3(0)	0(0)
Xenobiotics biodegradation & metabolism	11(3)	6(0)	3(2)	1(0)	1(1)

Numbers in brackets correspond to the number of reactions unique to the pathway, as an indicator of its taxon specificity.

### The distribution of phylogenetic groups within pathways

The metabolic pathways in KEGG are classified into 11 super-pathway categories. As a first step, we have summarized the distribution of reactions from the different phylogenetic groups within each super-pathway (Table 1). The most conserved pathways (where at least 80% of the reactions are universal) are those involved in the metabolism of nucleotides and several amino acids (including glutathione and selenoamino acid). The most recent pathways (in which most of the reactions have evolved after the emergence of the eukaryotic cell) are those involved in glycan biosynthesis and metabolism (84% of the reactions in eukaryotes – including metazoa and vertebrates; 16% universal) and in the metabolism of complex lipids (61% of the reactions in eukaryotes – including metazoa and vertebrates; 39% universal) (Table 1). The large number of vertebrata proteins involved in the metabolism of lipids was previously shown, and was related to the participation of these reactions in the biosynthesis hormones [19].

The following step aimed to investigate in detail the classification of reactions into the actual pathways. In total, 78 pathways were selected according to the criteria described in the **Methods **section, listed in Table 2. The pathways are grouped according to the classification of their most ancient unique reactions in order to have an estimate of their lineage of origin. As can be seen in Table 2, most pathways are composed of reactions present in more than one phylogenetic group, where a significant fraction of eukaryotic, metazoan and vertebrate-specific reactions (66%, 51% and 40% respectively) are integrated into pathways that have a universal origin, rather than lineage-specific ones. Only four of the pathways with pre-eukaryotic origin are composed solely of universal reactions (pentose phosphate pathway, glutathione metabolism, selenoamino acid metabolism, and methane metabolism). The diverse composition of most pathways is compatible with previous studies indicating that the KEGG pathways are not necessarily a single evolutionary unit, and in many cases they can be divided into several conserved modules acting as both evolutionary and functional units [20].

**Table 2:** The distribution of human reactions within metabolic-pathways.

Lineage of origin	Super pathway	Pathway (pathway identifier)	T	U	E	M	V
**Pre- eukaryotic origin (universal pathways)**	Carbohydrate metabolism	Glyoxylate & dicarboxylate metabolism (00630)	11(3)	9(3)	1(0)	0(0)	1(0)
		Galactose metabolism (00052)	15(3)	11(1)	1(0)	0(0)	3(2)
		Fructose & mannose metabolism (00051)	17(11)	11(6)	4(3)	1(1)	1(1)
		Pentose phosphate pathway (00030)	16(6)	16(6)	0(0)	0(0)	0(0)
		Aminosugars metabolism (00530)	17(12)	11(9)	4(2)	1(0)	1(1)
		Pyruvate metabolism (00620)	21(5)	20(5)	1(0)	0(0)	0(0)
		Butanoate metabolism (00650)	17(3)	14(1)	1(1)	1(0)	1(1)
		Glycolysis/Gluconeogenesis (00010)	27(5)	22(3)	2(0)	2(2)	1(0)
		Starch &sucrose metabolism (00500)	23(8)	15(6)	3(1)	1(0)	4(1)
		Citrate cycle (TCA cycle) (00020)	16(1)	14(1)	2(0)	0(0)	0(0)
	Energy metabolism	Sulfur metabolism (00920)	8(3)	3(1)	1(1)	0(0)	4(1)
		Oxidative phosphorylation (00190)	8(4)	7(3)	1(1)	0(0)	0(0)
		Nitrogen metabolism (00910)	9(1)	8(1)	1(0)	0(0)	0(0)
		Methane metabolism (00680)	5(1)	5(1)	0(0)	0(0)	0(0)
	Lipid metabolism	Biosynthesis of steroids (00100)	17(11)	9(5)	6(4)	0(0)	2(2)
		Fatty acid metabolism (00071)	17(1)	14(1)	2(0)	0(0)	1(0)
		Linoleic acid metabolism (00591)	5(1)	2(1)	1(0)	0(0)	2(0)
		Glycerolipid metabolism (00561)	16(4)	12(2)	3(2)	0(0)	1(0)
		Glycerophospholipid metabolism (00564)	23(9)	9(3)	11(3)	1(1)	2(2)
		Ether lipid metabolism (00565)	7(2)	2(1)	5(1)	0(0)	0(0)
		Fatty acid biosynthesis (00061)	11(9)	7(6)	4(3)	0(0)	0(0)
	Nucleotide metabolism	Purine metabolism (00230)	51(24)	41(20)	8(4)	0(0)	2(0)
		Pyrimidine metabolism (00240)	34(14)	29(13)	3(0)	0(0)	2(1)
	Amino acid metabolism	Methionine metabolism (00271)	13(2)	11(2)	1(0)	0(0)	1(0)
		Histidine metabolism (00340)	14(4)	11(3)	1(0)	0(0)	2(1)
		Arginine &proline metabolism (00330)	19(5)	15(4)	2(0)	1(1)	1(0)
		Lysine degradation (00300)	19(7)	10(1)	4(2)	1(1)	4(3)
		Urea cycle & metabolism of amino groups (00220)	21(7)	17(7)	3(0)	0(0)	1(0)
		Valine, leucine & isoleucine degradation (00280)	23(7)	19(5)	0(0)	0(0)	4(2)
		Tryptophan metabolism (00380)	25(11)	16(4)	4(4)	0(0)	5(3)
		Cysteine metabolism (00272)	7(1)	6(1)	1(0)	0(0)	0(0)
		Phenylalanine, tyrosine &tryptophan biosynthesis (00360)	6(1)	5(1)	1(0)	0(0)	0(0)
		Glycine, serine &threonine metabolism (00260)	31(10)	24(7)	2(0)	2(2)	3(1)
	Metabolism of other amino acids	Glutathione metabolism (00480)	10(1)	10(1)	0(0)	0(0)	0(0)
		Selenoamino acid metabolism (00450)	10(2)	10(2)	0(0)	0(0)	0(0)
	Glycan biosynthesis & metabolism	N-Glycan biosynthesis (00510)	21(3)	2(1)	13(2)	4(0)	2(0)
	Metabolism of cofactors & vitamins	Vitamin B6 metabolism (00750)	5(3)	3(2)	0(0)	0(0)	2(1)
		Folate biosynthesis (00790)	8(6)	6(4)	2(2)	0(0)	0(0)
		Riboflavin metabolism (00740)	5(2)	3(2)	0(0)	1(0)	1(0)
		Biotin metabolism(00780)	5(5)	1(1)	3(3)	1(1)	0(0)
		Porphyrin & chlorophyll metabolism (00860)	16(12)	13(10)	1(1)	1(0)	1(1)
		Nicotinate & nicotinamide metabolism (00760)	13(7)	7(4)	0(0)	0(0)	6(3)
		One carbon pool by folate (00670)	17(4)	15(2)	1(1)	1(1)	0(0)
		Pantothenate & CoA biosynthesis (00770)	12(6)	10(5)	0(0)	0(0)	2(1)

**Eukaryotic origin**	Carbohydrate metabolism	Inositol phosphate metabolism (00031)	16(3)	2(0)	11(2)	1(1)	2(0)
	Lipid metabolism	C21-Steroid hormone metabolism (00140)	10(4)	0(0)	2(1)	0(0)	8(3)
		Androgen &estrogen metabolism (00150)	13(2)	1(0)	3(1)	1(0)	8(1)
		Arachidonic acid metabolism (00590)	20(12)	4(0)	6(5)	0(0)	10(7)
		Fatty acid elongation in mitochondria (00062)	6(2)	3(0)	2(2)	0(0)	1(0)
		Sphingolipid metabolism (00600)	19(11)	6(0)	7(5)	1(1)	5(5)
		Bile acid biosynthesis (00120)	12(2)	3(0)	3(2)	0(0)	6(0)
	Amino acid metabolism	Alanine &aspartate metabolism (00252)	21(2)	18(0)	3(2)	0(0)	0(0)
		Tyrosine metabolism (00350)	20(4)	9(0)	3(1)	2(1)	6(2)
	Glycan biosynthesis & metabolism	Glycan structures – biosynthesis 1 (01030)	43(1)	1(0)	12(1)	14(0)	16(0)
		Glycosylphosphatidylinositol(GPI)- anchor biosynthesis (00563)	3(1)	0(0)	3(1)	0(0)	0(0)
	Metabolism of cofactors & vitamins	Retinol metabolism (00830)	3(3)	0(0)	1(1)	1(1)	1(1)
	Xenobiotics Biodegradation & metabolism	Metabolism of xenobiotics by cytochrome P450 (00980)	7(2)	3(0)	3(2)	1(0)	0(0)

**Metazoan- origin**	Metabolism of other amino acids	Taurine &hypotaurine metabolism (00430)	5(1)	2(0)	1(0)	1(1)	1(0)
	Glycan biosynthesis & metabolism	Heparan sulfate biosynthesis (00534)	7(1)	0(0)	0(0)	5(1)	2(0)

**Vertebrate- origin**	Carbohydrate metabolism	Nucleotide sugars metabolism (00520)	6(0)	5(0)	0(0)	0(0)	1(0)
		Pentose & glucuronate interconversions (00040)	7(1)	5(0)	0(0)	1(0)	1(1)
		Propanoate metabolism (00640)	15(0)	13(0)	1(0)	0(0)	1(0)
	Amino acid metabolism	Lysine biosynthesis (00300)	5(0)	2(0)	2(0)	0(0)	1(0)
		Phenylalanine metabolism (00360)	9(0)	6(0)	2(0)	0(0)	1(0)
	Metabolism of other amino acids	beta-Alanine metabolism (00410)	14(0)	11(0)	2(0)	0(0)	1(0)
	Glycan biosynthesis & metabolism	Glycan structures – degradation (01032)	18(0)	10(0)	2(0)	0(0)	6(0)
		Glycosphingolipid biosynthesis – globoseries (00603)	10(1)	2(0)	1(0)	1(0)	6(1)
		Glycosaminoglycan degradation (00531)	11(0)	4(0)	1(0)	0(0)	6(0)
		Glycosphingolipid biosynthesis – lactoseries (00601)	7(0)	0(0)	1(0)	0(0)	6(0)
		Keratan sulfate biosynthesis (00533)	5(0)	0(0)	0(0)	2(0)	3(0)
		Glycosphingolipid biosynthesis – neo-lactoseries (00602)	10(0)	0(0)	1(0)	2(0)	7(0)
		Glycan structure – biosynthesis 2 (01031)	22(0)	1(0)	3(0)	2(0)	16(0)
		O-Glycan biosynthesis (00512)	6(0)	0(0)	0(0)	1(0)	5(0)
		Glycosphingolipid biosynthesis – ganglioseries (00604)	8(0)	2(0)	0(0)	0(0)	6(0)
		Chondroitin sulfate biosynthesis (00532)	10(0)	0(0)	0(0)	4(0)	6(0)
	Biosynthesis of secondary metabolites	Limonene & pinene degradation (00903)	5(0)	2(0)	0(0)	0(0)	3(0)
		Monoterpenoid biosynthesis (00902)	3(0)	0(0)	0(0)	0(0)	3(0)
	Xenobiotics biodegradation & metabolism	gamma-Hexachlorocyclohexane degradation (00361)	5(1)	4(0)	0(0)	0(0)	1(1)

Numbers in brackets indicate the number of reactions unique to the pathway, as in Table 1. The pathways are ordered according to their lineage of origin, estimated according to the most ancient unique reaction. T – total; U – universal; E – eukaryotic-specific; M – metazoan-specific; V – vertebrate-specific.

### A characterization of lineage-specific innovations in the metabolic pathway repertoire

Of the 78 metabolic pathways identified in human, 44 pathways, more than half (56%), have a universal origin (Table 2). These universal pathways include mostly pathways involved in sugar, nucleotide, amino-acid, cofactor and energy metabolism. Such pathways were previously defined as a metabolic "skeleton", common to all domains of life [18,20,21]. Not all of the amino acid metabolism pathways can be classified as universal and many of them are classified as specific to a more recent lineage, demonstrating the profound differences in amino acid metabolism between mammals and bacteria [22].

Pathways which appear to have a eukaryotic origin include pathways which are involved in the biosynthesis of characteristic components of the eukaryotic membrane, such as sphingolipids or glycan structures, or pathways involved in inter- and intra-cellular signaling (retinol metabolism, inositol phosphate metabolism, GPI anchor biosynthesis). Many other pathways of a putative eukaryotic origin are involved in processes which are characteristic of higher taxa including animals, such as androgen and estrogen metabolism, C21-steroid hormone metabolism, and bile acid biosynthesis. In these pathways, the majority of reactions belong to more recent lineage groups, and only a few reactions are specific to eukaryotes (Table 2).

Pathways which have metazoan or vertebrate origin have evolved after the transition of the unicellular ancestor of animals to multicellularity. It is therefore unsurprising that many of them are involved in tissue-specific activities. Examples for such activities include neuronal guidance and differentiation (ganglioside biosynthesis), absorption of ingested lipids in the gastrointestinal track (taurine metabolism), cartilage differentiation (chondroitin sulfate and kertan sulphate biosynthesis), and blood cell recognition (glycosphingolipid biosynthesis). The evolution of these eukaryotic-, metazoan-, and vertebrate-specific pathways and their functional significance is discussed below.

### The evolution of the metabolic network in humans

**Figure 1:**
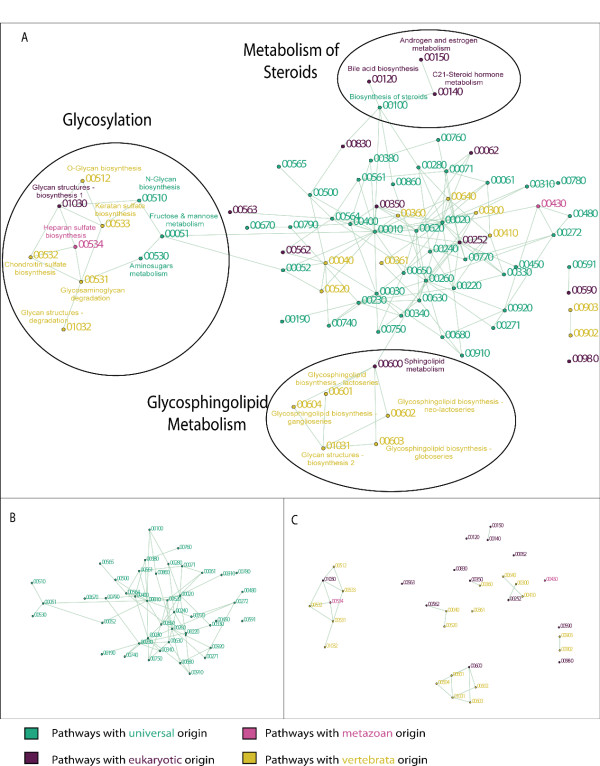
**A network representation of human metabolic pathways.** Layout and network construction were performed using the Biolayout software [33]. Each node represents one of the pathways from **Table 2**, the links between the nodes were retrieved from the KEGG database diagrams. The full names of the pathways are listed in **Table 2**. (A) The complete network; (B) Universal pathways; (C) The eukaryotic-, metazoan- and vertebrate-specific pathways – universal pathways are omitted for clarity.

The human pathways were linked to form a network (see Methods section) where each pathway is a node. The constructed network is shown in Figure 1. Almost all the pathways, namely 73 out of 78, are linked to form a single connected network. The core of the network is mostly formed by universal pathways, where more recent pathways are in many cases added as peripheral extensions. The centrality of the universal pathways is also indicated by their average connectivity (4.8 links per pathway), which is higher than the average connectivity of pathways with a more novel origin (3.2, 3.5, and 3.4, for pathways with eukaryotic, metazoan and vertebrate origin, respectively).

With the exception of two pathways (linoleic acid metabolism and selenoamino acid metabolism), all universal pathways form a connected component of the network (Figure 1B). The most highly-connected nodes represent pathways involved in carbohydrate metabolism: the glycolysis/gluconeogenesis pathway, TCA cycle, and pyruvate metabolism (20, 17, and 13 edges respectively). Other highly-connected nodes (at least 10 edges) are two amino-acid synthesis pathways (alanine and aspartate metabolism, glycine serine and threonine metabolism).

When constructing a network only from the lineage-specific pathways, one can observe that, unlike the case of universal pathways, lineage-specific pathways do not form a single-component network (Figure 1C). The lineage-specific pathways are clustered into seven single-node components and seven networks where the number of nodes ranges between two and seven.

**Figure 2:**
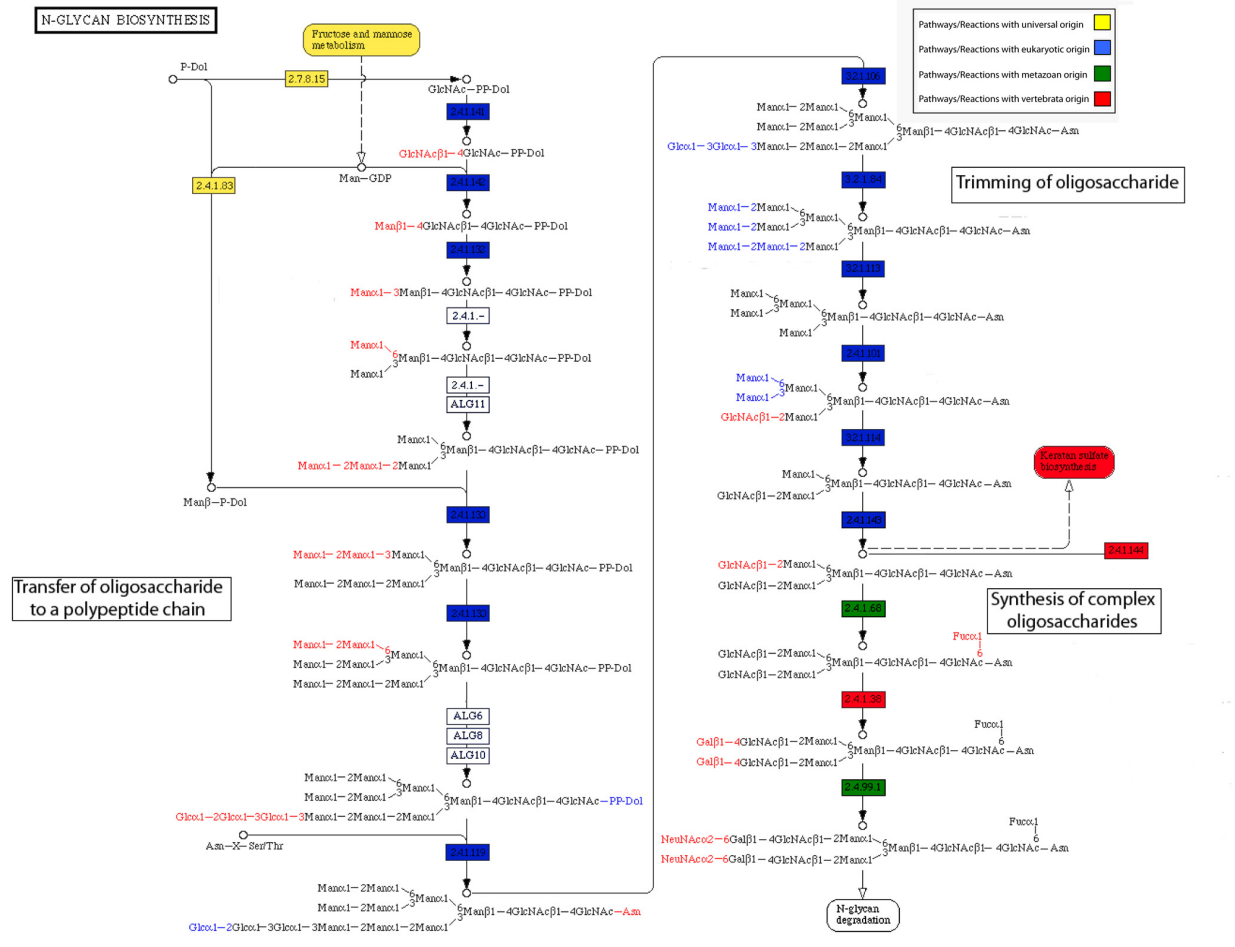
**Phylogenetic structure of the N-glycan biosynthesis pathway.** The diagram was constructed according to information in the KEGG database. Each box represents a reaction and each oval box represents a pathway. The colors represent the estimated phylogenetic origin of the reactions and pathways (as listed in **Table 2**).

**Figure 3:**
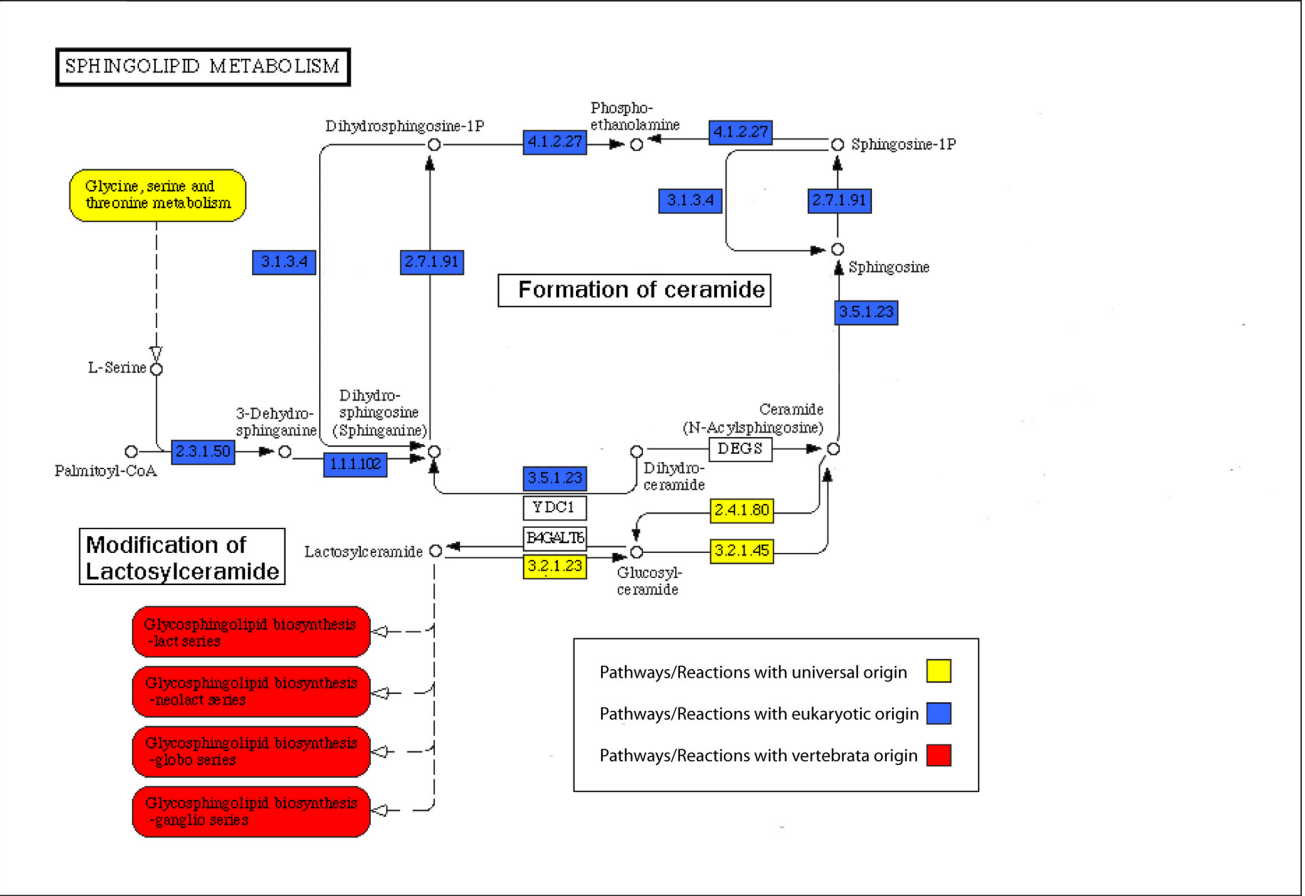
**Phylogenetic structure of the sphingolipid metabolism pathway.** Diagram construction and display conventions as in **Figure 2**.

**Figure 4:**
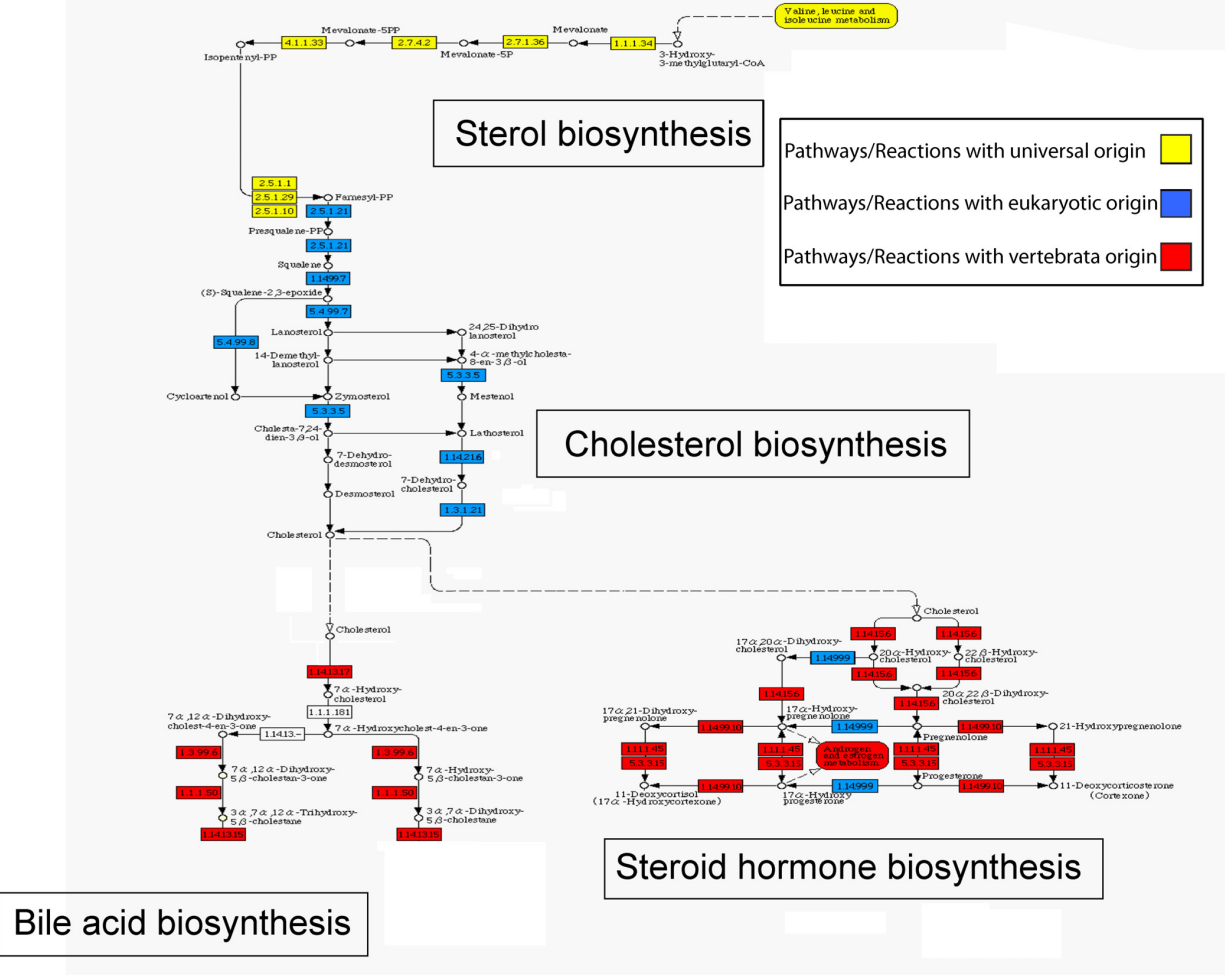
**Phylogenetic structure of the cholesterol biosynthesis pathway.** Diagram construction and display conventions as in **Figure 2**.

Our analysis demonstrates that the conserved core of metabolism, reported to be common to all domains of life, forms a structural core component of the metabolic network (the universal pathways in Figure 1). Within the formed network, we observe that lineage-specific pathways are added to the core component of the network in two characteristic ways: with direct links, or the formation of peripheral extensions. Lineage-specific pathways which are involved in amino-acid metabolism are in all cases linked directly to the universal-core network. Lineage-specific pathways which are involved in lipid metabolism and glycan biosynthesis are in most cases clustered into a few network components which are added as external extensions to the core network of universal pathways (the sub-networks are marked in Figure 1A). Since the latter have a significant contribution to the appearance of mammalian-specific phenotypes, we have studied in detail the structure of the network formed by the reactions from these pathways. As discussed below (and demonstrated in Figures 2, 3, 4), in all these sub-networks the phylogenetic view of the pathway underlines how gradual accumulation of reactions corresponds to the appearance of lineage-specific phenotypes. The significance of these pathways to the emergence of the eukaryotic cell and the appearance of multicellular animals is further discussed below.

#### Glycosylation

The classification approach taken here has assigned the N-glycan biosynthesis pathway as universal. This pathway leads to more recent, lineage-specific pathways (Figure 1A), which are involved in the biosynthesis of glycoproteins with specialized functions in specific vertebrate tissues. The classification of the pathways correspond with current knowledge, where the glycosylation of proteins is considered to have originated in a prokaryotic, pre-compartalized, ancestor and have gradually evolved into a multi-stages, tissue-specialized process. The individual reactions and their phylogenetic origins are shown in greater detail in Figure 2. The evolution of the pathway provides an example for the contribution of the compartalization of the eukaryotic cell to the appearance of novel extracellular molecules, and to their further specification in the tissues of high-animals.

Glycosylation is the attachment of a carbohydrate residue to a protein [23], and it is the most frequent post-translational modification in eukaryotic species [24]. In mammals, the glycosylation of proteins begins in the endoplasmic reticulum (ER) with the synthesis of a large oligosaccharide residue (Glc_3_Man_9_GlcNAc_2_), which is transferred to nascent polypeptide chains (Figure 2). Following its transfer, Glc_3_Man_9_GlcNAc_2 _undergoes trimming of the glucose and some of the mannose residues, first in the ER and then in the Golgi, in order to build it up again with different sugars to form complex N-glycans [25].

The evolutionary origin of the mammalian glycosylation can illuminate the significance of this complex process. The synthesis of N-linked glycans (which takes place in the ER) stems from homologous processes in the plasma membrane of archaea or bacteria [24]. The early stages of glycosylation are homologous between prokaryotes and eukaryotes where both use similar mechanisms to transfer the oligosaccharide residue (yellow boxes in Figure 2). In contrast, the identity of the oligosaccharide residue is not conserved between eukaryotes and prokaryotes. Whereas prokaryotic species exhibit a great diversity with regard to the oligosaccharide used, almost all eukaryotes transfer the same structure – Glc_3_Man_9_GlcNAc_2_.

In most eukaryotic species, the early steps in N-glycan processing in the ER are conserved. The addition of further saccharides to the ancestral oligosaccharide during eukaryotic evolution is probably driven by the internalization of glycoprotein biosynthesis from the plasma membrane to the ER and the concomitant need to export newly synthesized proteins to the cell surface [24]. Later modifications of the carbohydrate groups (green/red boxes in Figure 2) exhibit considerable diversity between species and cell types [25], whereas the switch from the use of oligomannose (as in yeast) to complex N-glycans (as in mammals) correlates with the appearance of multicellular organisms [26].

The importance of complex N-glycans in the evolution of multicellular species might be related to the role of glycosaminoglycans in the formation of the extracellular matrix. Though, as can be predicted from their great abundance and structural variety, the functions of proteoglycans in vertebrates are far more diverse. The main groups of glycosaminoglycans in vertebrates play different roles in different tissues: Keratan sulfate and chondroitin sulfate are important structural components of connective tissues (cartilage and bone); Heparan sulfate regulates hematopoietic processes; Dermatan sulfate is found mostly in skin [27].

#### Glycosphingolipid (GSL) metabolism

In the network of pathways (Figure 1A), the eukaryotic pathway of sphingolipid metabolism is linked to the core network via the universal serine metabolism pathway. The sphingolipid metabolism pathway leads to vertebrate-specific pathways, which are involved in the metabolism of cell-type specific GSLs – the metabolism of gangliosides which are characteristic of nerve cells, and the metabolism of the lactosylceramide series which are characteristic of groups of blood cells. The classification of pathways corresponds with current knowledge, and provides an example for an eukaryotic innovation (novel membrane component) which gave rise to the development tissue-specialized characteristics.

Glycolipids are sugar-containing lipids found in the membrane of cells, where in eukaryotic cells, glycolipids are derived from sphingosine (blue boxes in Figure 3) [28]. The variation in type, number, linkage and further modification of the sugar residues give rise to a combinatorial variety of GSLs [29]. Different GSL series are characteristic of different animals, where lactosylceramide is the common precursor for the GSL series found in vertebrates (red boxes in Figure 3). Different series of lactosylceramide-derived GSLs have their unique expression patterns in specific cell types, where blood cells and nerve cells, in particular, have a characteristic composition of GSLs [30].

#### Biosynthesis of cholesterol

In the network of pathways (Figure 1A), the universal pathway of sterol biosynthesis leads to pathways that are involved in process which are characteristic of different tissue types. The synthesis of cholesterol and its derivates provides an additional example for a novel eukaryotic membrane component which in higher animals is used as a precursor for the synthesis of specialized molecules.

Cholesterol is a lipid present in the membrane of eukaryotes, but not in the membrane of most prokaryotes. In animals, cholesterol is also a precursor of many signaling molecules including steroid hormones and bile acids.

The synthesis of cholesterol is illustrated in Figure 4. In prokaryotes, the synthesis of sterol had stopped at squalene [31]. In this ancient pathway (partly represented by the yellow boxes in Figure 4), which is assumed to have evolved before the appearance of oxygen in the atmosphere (and before the appearance of eukaryotes), squalene is hydrated to form hopanoid – a sterol-like molecule found in the membrane of prokaryote species. Once aerobic conditions developed, the oxidation of squalene by O_2 _gave rise to the formation of genuine precursors of sterols – lanosterol in vertebrates and fungi (blue boxes in Figure 4), and cyclortenol in plants. The degradation of those precursors to form sterols may have paved the way towards the eukaryotic membrane, with its efficient combination of n-acyl chains and sterol [31].

Derivatives of cholesterol, such as steroid hormones and bile acid (red boxes in Figure 4), have roles, which are specific to vertebrates. Bile acids are polar derivatives of cholesterol which facilitate the absorption of lipids in the small intestine. Cholesterol is also the precursor of the major classes of steroid hormones, which are regulators of different processes across tissues, including the development of sex characteristics, and the degradation of fat [28].

## Discussion

Here, we describe a function-based analysis of the evolution of human metabolic properties. The main novelty in this analysis is the provision of a global view on the evolution of the mammalian network. While previous studies have characterized the metabolic "skeleton", common to all domains of life, or innovations characteristic of one of the lineages leading to mammals [18,19,21], a global view of the metabolic innovations in mammals was not previously reported. Therefore, although the phyletic classification of pathways is in most cases in agreement with biochemical literature, this study provides for the first time a comprehensive analysis of the origin of the complement of the mammalian metabolic pathways. Furthermore, this study not only concerns the characterization of 'ancient' and 'novel' pathways but also describes for the first time the way they are integrated to form a metabolic network. With the use of pathway maps, it is possible to view the position of lineage-specific pathways in the biochemical network (i.e., the phylogenetic structure of the network). Thus, it is possible to uncover the core reactions of pathways, and the additional inventions during evolution, in a manner consistent with current biological knowledge. This general approach is applicable to any species and more complex phylogenies; the analysis also suggests ways by which this type of evolutionary information can be incorporated into future metabolic databases.

However, the limitations of such an approach should also be underlined. First, the observations reported here are highly dependent on the conventions of the reference database (in this case, the KEGG resource). The classification of reactions into pathways is somewhat subjective, since it can be implemented in a varied manner. Second, the characterization of the set of reactions in species, as well as the classification of reactions into phylogenetic groups is dependent both on the sensitivity of the homology assignment procedure and the range of species analyzed. Third, this analysis concerns only a limited set of reactions whose biochemical function and exact position in the metabolic network are well defined (fully-assigned EC reactions). Such information is not available for a substantial part of the lineage specific reactions which were therefore not considered in this work. These performance issues affect our ability both to reconstruct the complement of the reaction set and to delineate the contribution of each lineage towards the development of the metabolic repertoire during evolution. Yet, despite these limitations, the findings of this analysis are entirely consistent with current knowledge (as shown in [13] and here); consequently, it is reassuring that the general picture of gradual evolution with a patchwork of metabolic innovations related to the physiological needs and capabilities of the corresponding taxa is very accurate. Moreover, the classification of reactions into phylogenetic reaction sets is generally in agreement with classifications obtained using different procedures [15]. Finally, as discussed below, the structural organization of the metabolic network provides a valuable insight on the gradual evolution of phenotypes which are characteristic of the lineages studied.

Our analysis indicates that a universal metabolic core vertically inherited from a pre-eukaryotic ancestor remained highly conserved all the way up to mammals. Compatible with previous studies which have characterized a universal metabolic 'skeleton' [18,20,21], the universal pathways in humans are mainly involved in the metabolism of the basic building blocks of every living cell: carbohydrates, lipids, and amino-acids. Only a limited number of reactions and pathways seem to be specific to eukaryotes, metazoa and vertebrates. The eukaryotic-specific reactions are in many cases involved in the metabolism of the complex structures found in the membrane of the eukaryotic cells, or the membranes of cellular organelles. In metazoa, these reactions catalyze the first steps in the synthesis of extracellular molecules, primarily those abundantly found in multicellular organisms [32]; extracellular proteins participate in all animals in inter-cellular communication and cell adhesion. In reactions specific to vertebrates, the extracellular molecules provide precursors for the biosynthesis of more complex molecules, whose function is in many cases characteristic of a specific cell type or tissue.

## Conclusion

The phylogenetic-structure of the metabolic network, which is described here for the first time, emphasizes the gradual evolution of processes which are specific to a tissue, whereas the development of a phenotype in an ancient lineage provides a platform for the evolution of more recent traits. As the phylogenetic representation of the metabolic network puts gene innovations within an evolutionary context, it demonstrates in a quasi-quantitative manner the importance of events such as the development of the eukaryotic membrane, cellular organelles and the system of transport between them, towards the emergence of multicellular life. While the basic mechanisms of inter-cellular communication are common between invertebrates and vertebrates, many of the vertebrate-specific reactions are involved in tissue-specific functions. The evolution of these pathways must have coincided with the emergence of cell types and organs.

Finally, this network approach highlights evolutionary junctions, at which ancestral species gained the ability to catalyze a new reaction type or use a new substrate. Examples of such junctions from the analysis performed here are cholesterol and sphingiosine, molecules whose biosynthesis and function are lineage-specific. The study of such junctions can contribute towards our understanding of the co-evolution between enzymes and substrates, and the mechanisms of biochemical innovation during evolution.

## Authors' contributions

SF conceived and performed the analysis, and drafted the versions of the manuscript. LG assisted in the reconstruction and analysis of the network, and contributed to preparation of the final manuscript. CAO assisted in the organization and writing of the manuscript and contributed with critical review. JMT contributed with discussion of the draft versions and critical review. All authors read and approved the manuscript.
